# Residential green space, walkability, and cardiometabolic biomarkers in midlife women: a longitudinal cohort study

**DOI:** 10.1088/2752-5309/ae61e7

**Published:** 2026-05-15

**Authors:** Seema Desai, Xiangmei (May) Wu, Jinshil Hyun, Carol A Derby, L Elaine Waetjen, Bradley M Appelhans, Sung Kyun Park, Keita Ebisu

**Affiliations:** 1 Air and Climate Epidemiology Section, Office of Environmental Health Hazard Assessment, California Environmental Protection Agency, Oakland, CA, United States of America; 2 Department of Neurology, Albert Einstein College of Medicine, Bronx, NY, United States of America; 3 Department of Epidemiology and Population Health, Albert Einstein College of Medicine, Bronx, NY, United States of America; 4 Department of Obstetrics and Gynecology, School of Medicine, University of California Davis, Davis, CA, United States of America; 5 Department of Family and Preventive Medicine, Rush University Medical Center, Chicago, IL, United States of America; 6 Departments of Epidemiology and Environmental Health Sciences, School of Public Health, University of Michigan, Ann Arbor, MI, United States of America

**Keywords:** green space, walkability, cardiovascular health, midlife women, menopausal transition, longitudinal modeling

## Abstract

*Background*. Cardiovascular disease remains the leading cause of death among U.S. women. Green spaces and neighborhood features may influence cardiometabolic health by modulating environmental stressors and physical activity. Few longitudinal studies have examined green space, walkability, and cardio-inflammatory biomarkers, particularly in midlife women during the menopausal transition—a period of elevated cardiovascular risk. *Methods*. We analyzed longitudinal data from the multi-ethnic, multi-site U.S. Study of Women’s Health Across the Nation (1999–2017) to examine associations between residential green space, walkability index, and cardio-inflammatory biomarkers across multiple clinical visits. Green space exposure was assessed using the National Land Cover Database (NLCD), with supplemental measures from the Normalized Difference Vegetation Index (NDVI). Exposures, categorized into tertiles, were weighted by 12 month geocoded residential history. Linear mixed-effects models estimated associations with systemic inflammatory, fibrinolytic, and lipid biomarkers, adjusting for socioeconomic, lifestyle, and environmental factors. Interaction analyses tested effect modification, with subgroup effects estimated. *Results.* Medium residential NLCD greenness in 1000 m buffer was associated with an 11.5% lower (95% Confidence Interval: −18.4, −3.9%) high-sensitivity C-reactive protein (hs-CRP), along with lower fibrinogen and tissue-plasminogen activator antigen levels compared to low greenness tertile, suggesting anti-inflammatory and anticoagulant effects. Higher NDVI greenness showed a similar negative association with hs-CRP (−10.2%, 95% CI: −18.6, −1.1%). Compared to low walkability tertile, medium walkability group was linked to higher low-density lipoprotein and total cholesterol. Effect modification analyses indicating lower hs-CRP, higher high-density lipoprotein (HDL), and lower triglycerides, suggested potential biological interactions between green space and women who transitioned to post-menopausal period. *Conclusion*. Findings support green space’s role in reducing systemic inflammation while highlighting the complex interplay of walkability, air pollution, and urban factors. Greenness and walkability capture distinct built environmental aspects, underscoring the need for green infrastructure to mitigate gentrification, reduce pollution, and support cardiovascular health.

**Abbreviations**
BMI: Body mass index
CI: Confidence interval
CVD: Cardiovascular disease
HDL: High-density lipoprotein
hs-CRP: High-sensitivity C-reactive protein
IQR: Interquartile range
IL-6: Interleukin-6
LDL: Low-density lipoprotein
M: Meter
NDVI: Normalized difference vegetation index
NLCD: National land cover database
PM_2.5_: Fine particulate matter
SES: Socioeconomic status
SWAN: Study of Women’s Health Across the Nation
TC: Total cholesterol
TG: Triglycerides
tPA-ag: Tissue-type plasminogen activator antigen

## 1. Introduction

CVD remains the leading cause of death in the United States (U.S.), with coronary heart disease accounting for 39.5% of CVD-related deaths, followed by stroke (17.6%), other CVD (17.0%), and hypertensive diseases (14.0%) [1]. The American Heart Association projects that by 2030, 43.9% of U.S. adults will be affected by CVD [2]. Much of the CVD burden is attributable to modifiable factors such as smoking, poor diet, physical inactivity, inadequate sleep, and related metabolic conditions [3]. These factors contribute to systemic inflammation, endothelial dysfunction, and lipid dysregulation that drive atherosclerosis and vascular remodeling. Importantly, these risks develop within broader social and neighborhood contexts that shape long-term cardiovascular vulnerability.

Residential land use is an important contextual factor, as built environment features—including green space and neighborhood walkability—have been associated with physical, mental, and cardiometabolic health, with green space linked to lower systemic inflammation and reduced cardiovascular mortality through pathways such as improved air quality, stress reduction, and increased opportunities for physical activity and social interaction [4]. Gascon *et al* reported that higher residential greenness was generally associated with lower cardiovascular mortality, though findings varied across study designs, populations, and greenness measures [5]. Similarly, James *et al* observed lower all-cause and cancer mortality with greater greenness in a large U.S. cohort of female nurses, but no association with cardiovascular mortality, and the study relied on a single greenness metric in a largely homogeneous population [6].

Walkable neighborhoods have similarly been associated with favorable lipid profiles and lower CVD risk, likely through increased active transportation and daily physical activity [7–9]. However, these associations are not uniform, as higher walkability may also coincide with greater density, traffic exposure, and socioeconomic gradients contributing to inconsistent associations with cardiometabolic biomarkers [10, 11].

Green space and walkability are interrelated yet structurally distinct dimensions of the built environment [8, 10, 12]. In some settings, parks and pedestrian-oriented design may align greenness with walkability; in others, denser mixed-use areas may contain less vegetation and higher pollution levels, producing inverse spatial patterns. Because these features may operate through overlapping yet distinct pathways, it is important to consider their unique and shared associations when evaluating cardiometabolic health.

These environmental influences may be particularly relevant during the menopausal transition, a period characterized by hormonal changes that increase systemic inflammation, lipid disruptions, and vascular dysfunction [13–17]. Air pollution and other neighborhood exposures have been linked to heightened inflammatory and hemostatic responses in midlife women, suggesting that the built environment may interact with biological vulnerability during this life stage [18–20]. Beyond environmental exposures, lower SES amplifies stressors and restricts health-promoting behaviors, exacerbating health disparities. These findings highlight the need to consider both environmental and social determinants in built environment research [21, 22].

Despite growing evidence, most prior studies have assessed greenness at single time points, limiting insight into longitudinal associations with cardiometabolic biomarkers [23, 24]. Research using comprehensive, time-weighted greenness measures in U.S. midlife women remains limited, and greenness metrics such as NDVI and NLCD have rarely been evaluated in parallel to capture multidimensional exposure.

This study leverages repeated measures from the SWAN to examine associations between time-weighted green space metrics, neighborhood walkability, and cardio-inflammatory biomarkers across midlife. We hypothesize that higher levels of residential green space are associated with a long-term benefit on cardiovascular health among midlife women. We also explored whether neighborhood walkability may be associated with these health endpoints. By focusing on midlife women, a key period of menopausal transition, this research provides valuable insights into the long-term relationship between health impacts and residential land use in this at-risk population, informing urban planning and public health strategies.

## 2. Methods

### 2.1. Population

SWAN is a multi-center, multi-ethnic, community-based prospective cohort study on health patterns during the menopausal transition in U.S. midlife women. At baseline, 3,306 women (aged 42–52) were recruited from seven sites (Chicago IL, Detroit MI, Los Angeles CA, Newark NJ, Oakland CA, Pittsburgh PA, and Boston MA). Non-Hispanic White women were enrolled at all sites, with specific racial/ethnic groups recruited at certain sites: African American (Chicago, Pittsburgh, Detroit, Boston), Chinese (Oakland), Japanese (Los Angeles), and Hispanic (Newark). This site-specific recruitment strategy was designed to ensure adequate representation and statistical power within each racial/ethnic group while reflecting the demographic composition of the communities surrounding each clinical site. Recruitment details, including eligibility criteria and annual follow-up procedures, have been described in previous studies [18, 25]. This study analyzed data from six SWAN sites, excluding Boston due to unavailable geocoded residential data. Serum samples from clinical visits 3 (1999–2000), 5 (2001–2002), 7 (2003–2004), 9 (2005–2006), 12 (2010–2011), and 15 (2015–2017) were used. Observations with missing blood collection dates through visit 15 were removed. Additional exclusions across all study visits included 177 participants with cancer, stroke, or external-cause death, a small number who got pregnant or relocated outside the mainland U.S., and 655 participants with only one visit. The final analytic sample comprised 2,008 women (8,980 observations), with around 360 participants per site except for Newark, which had 195. The study protocol was approved by the institutional review board at each site, and all participants provided written informed consent during each study visit.

### 2.2. Outcome

Fasting blood samples were collected at each SWAN clinical visit, and biomarkers were assayed using protocols described in prior studies [26, 27]. Detailed assay methods are provided in the supplementary text S1. Assay methods, including instrumentation and calibration procedures, varied across visits reflecting protocol updates over the study period. The inflammatory and hemostatic markers analyzed in this study included hs-CRP, blood lipids such as TC, HDL, LDL, and TG, along with fibrinogen, endothelin-1, IL-6, and tPA-ag. hs-CRP and IL-6 are markers of systemic inflammation implicated in atherosclerotic plaque development and instability. LDL-cholesterol, TC, and TG reflect atherogenic lipid burden, whereas HDL-cholesterol is generally considered cardioprotective. Endothelin-1 is a potent vasoconstrictor associated with endothelial dysfunction. Fibrinogen is an acute-phase reactant involved in coagulation and thrombosis, and tPA-antigen reflects fibrinolytic system activity and endothelial activation [28–30]. All biomarkers were measured at visits 3, 5, and 7, with additional measurements available for two of the three subsequent visits (9, 12, or 15) for all biomarkers except for tPA-ag, which was only available through visit 7.

### 2.3. Exposure

Residential histories were geocoded for all SWAN participants, with addresses randomly displaced by up to 400 feet for confidentiality. Move dates were recorded, and when unavailable, approximated using midpoints between sampling dates or relevant timelines, such as mail communications.

Green space exposure was assessed using NLCD and NDVI. NLCD, derived from Landsat satellite imagery and managed by the Multi-Resolution Land Characteristics Consortium, captures multiple spectral bands into 16 land cover types (e.g. forests, wetlands, urban areas) at 30 m resolution, updated every five years across the U.S [31]. Exposure metrics were linked to geocoded residential addresses at each study visit. For each visit, 1000 m buffers around each residence were used to calculate the percentage of three land cover types classified as green space: grassland, shrubs, and forests (see supplementary material figure S1 for illustration). A sensitivity analysis using a 500 m buffer for NLCD was also conducted. Because NLCD data are available at 5 year intervals, greenness at each visit was assigned using the NLCD year closest to each participant’s residential history (e.g. 2001, 2006, 2011). For participants who did not move in the past 12 months from the date of visit, exposure was based on their address at the visit. For those who moved in the year before a study visit, greenness was weighted by the number of days spent at each address to reflect cumulative 12 month exposure.

Green space exposure was also assessed using NDVI, derived from the MOD13Q1 MODIS 16 d composite product, which provides vegetation indices at 250 m resolution with built-in compositing and quality assurance algorithms to minimize cloud, snow, and atmospheric artifacts [32]. NDVI, calculated as the ratio of visible red to near-infrared reflectance (−1–1, with higher values indicating denser vegetation), was available starting in February 2000; therefore, exposure was assessed from visit 5 onward. For each participant, we calculated a 12 month average of NDVI values prior to each visit, applying the same residential history weighting approach used for NLCD.

These two databases provided complementary green space measures: NLCD offers detailed land cover classifications, while NDVI provided a continuous and temporally resolved measure of vegetation density. Because NLCD is a categorical 30 m land cover product in which each pixel is assigned a single dominant class, larger buffers were applied to improve stability of aggregated land cover estimates in dense urban settings. In contrast, NDVI is a continuous vegetation index that captures sub-pixel greenness, allowing assessment at finer spatial scales. Continuous NLCD- and NDVI-based exposures were categorized into low, medium, and high tertiles to address the left-skewed distribution and enhance interpretability in non-linear models.

Walkability around each residential neighborhood was assessed using the walkability index at the census block group level from the U.S. Environmental Protection Agency Smart Location Mapping dataset [33]. The index of 1–20 integrates residential density, land use diversity, intersection density, and transit proximity, with higher values indicating more pedestrian-friendly neighborhoods. Like NLCD and NDVI, this index was weighted based on residential history and then categorized into low (⩽11.8), medium (11.8–14.7), and high (>14.7) tertiles in the analysis.

### 2.4. Covariates

At SWAN baseline, participants completed a questionnaire on demographics and SES, including age, self-reported race/ethnicity, and education level (high school or less, some college, or college graduate) [27]. At annual clinic visits, participants provided visit-specific data on medical history, psychosocial factors, lifestyle behaviors (smoking, alcohol, etc.), comorbidities (diabetes, stroke, cancer etc.), and any medication use.

Menopausal status was categorized as pre-menopausal (no change in bleeding patterns), early perimenopausal (some menstrual irregularities in the past year but intermenstrual interval less than 3 months), late perimenopausal (no bleeding in 3–11 months), post-menopausal (natural menopause with no bleeding in 12 months or hysterectomy with bilateral salpingo-oophorectomy), and unknown due to missing data on hormone therapy or hysterectomy status [17, 27, 34]. Income levels were consolidated into four groups: <$20 000, $20 000–<$49 999, $50 000–$99 000, and >$99 000. Alcohol consumption was classified as low (none or <1 serving/month), moderate (⩽1 week or 0.3 d), and high (⩾2 week or >0.3 d) [18]. Other covariates included visit numbers (visit 3, 5, 7, 9, 12, 15), BMI (<25, ⩾25 and <30, and ⩾30 kg m^−2^), age, education level, smoking status (yes/no), and depression status (yes/no). Any medication use was categorized as low (⩽1 d), medium (2–4 d), and high (>4 d).

PM_2_._5_ exposure was estimated using 1×1 km satellite-based grid data [35]. A geocoded address was assigned to the corresponding grid, and a past 12 month mean prior to the study visit was calculated by taking account moving history, similar to greenness exposures. These visit-specific 12 month mean PM_2_._5_ levels were categorized as low (<11.7 *μ*g m^−3)^, medium (11.7–14.9 *μ*g m^−3^) or high (>14.9 *μ*g m^−3^) tertiles. Additional SES covariates included population density (persons per square mile) and poverty ratio (percent living below twice the poverty threshold at census block group level), derived from the 2010 U.S. Census and 2006–2010 American Community Survey, respectively, to ensure temporal consistency with the SWAN cohort visits.

Race/ethnicity and education were considered time-invariant variables, while time-varying covariates included PM_2_._5_ exposure, visit number, age, BMI, menopausal status, smoking, alcohol consumption, income, depression, and any medication use.

Covariate selection was informed by a directed acyclic graph (DAG) representing hypothesized relationships between residential green space, cardiometabolic biomarkers, and demographic, behavioral, and neighborhood-level factors (supplementary figure S2) [36].

### 2.5. Statistical analyses

#### 2.5.1. Descriptive statistics

Baseline demographic, socioeconomic, lifestyle, and health-related characteristics were summarized using means and standard deviations for continuous variables and frequencies and percentages for categorical variables.

Median and IQR values were calculated for each biomarker across visits and menopausal stages. Percentiles (10th, 33rd, 50th, 67th, and 90th) were computed for exposures, including NLCD- and NDVI-based greenness, walkability index, and PM_2_._5_. A Spearman correlation matrix, using pairwise complete observations, was used to evaluate relationships among continuous exposure variables, including time-weighted NLCD green space metrics (1000 m and 500 m), NDVI 250 m buffer, walkability index, and PM_2_._5_concentration due to their skewed exposure distributions.

#### 2.5.2. Regression analysis

We used linear mixed-effects models to account for the hierarchical structure of repeated biomarker measures, incorporating random intercepts for site and participants nested within sites to address clustering and within-participant variability. Biomarker levels were log-transformed for normalization and analyzed with environmental predictors, based on exposures in the 12 months preceding each study visit and adjusting for covariates. The analysis included three primary indicators: tertiles from residential green space (NLCD-1000 m and NDVI-250 m buffers), and walkability index, each assessed as a separate exposure. It should be noted that NDVI-based analyses were restricted to visits 5 onward due to data availability; for tPA-ag, analyses included visits 5 and 7 only, where both exposure and outcome data overlapped.

Model assumptions were assessed using Q–Q plots for residual diagnostics to evaluate normality. Log-transformation improved variance stability for right-skewed biomarkers, and continuous and tertile specifications of green space yielded similar inferences. (supplementary figures S3.1 and S3.2.)

SES covariates were selected from income, population density, and poverty ratio using a stepwise approach based on the Bayesian information criterion due to their high correlation and overlapping constructs. Income was retained for optimal model fit, while population density and poverty ratio were excluded to maintain parsimony.

Measures of associations are presented as percentage changes in biomarker levels and corresponding 95% CIs, comparing the top two tertiles of each exposure variable to the bottom tertile.

#### 2.5.3. Effect measure modification

The association between NLCD-measured green space and cardio-inflammatory biomarkers was assessed by menopausal status (perimenopausal [early and late combined]: observations *n* = 2,596, postmenopausal: 5,127) and BMI categories (normal: 2901, overweight: 2880, and obese: 2245) using interaction analyses to account for hormonal and adiposity-related influences on inflammation [17, 37, 38]. The pre-menopausal group (*n* = 303) was excluded due to the small sample size. Since NDVI data was available only from visit 5, analyses were restricted to NLCD. Interaction models included an NLCD times modifier term to statistically assess effect modification, with likelihood ratio tests comparing models with and without interaction terms. Total effects, derived from interaction models, represent estimated biomarker changes within each subgroup, incorporating both the main exposure effect and its interaction with the modifier. These interaction analyses account for transitions in menopausal status (e.g. from pre- to peri-menopausal or peri- to post-menopausal) and changes in BMI status over time.

#### 2.5.4. Sensitivity analyses

To address potential confounding, one sensitivity analysis adjusted for walkability in models examining associations between green space (NLCD 1000 m and NDVI 250 m) and biomarkers. Another analysis restricted the model to 533 participants with biomarker data from all six visits (3,198 observations across visits 3, 5, 7, 9, 12, and 15). For NDVI-based green space, available from visit 5 onward, 563 participants contributed 2,815 observations (visits 5, 7, 9, 12, and 15). Furthermore, we examined NLCD green space metrics using a 500 m buffer for the full cohort. Based on the DAG, BMI and depression were considered potentially lying on the causal pathway linking green space to biomarkers. We therefore re-estimated models sequentially excluding these two variables and compared estimates with the primary model to evaluate changes in effect size. Lastly, we modeled green space (NLCD 1000 m), NDVI (250 m), and walkability as continuous variables scaled per IQR increase. PM_2_._5_ was included as a continuous covariate. These models were estimated using the same mixed-effects structure as the primary tertile analyses.

All analyses were conducted using the lme4 package for linear mixed-effects models in R (version 4.4.0) [39].

## 3. Results

The SWAN cohort comprised a diverse population of 2,008 midlife women with a mean age of 49.9 years (standard deviation; SD 3.1) at study visit 3. Nearly 45% of participants were White, with African American women being the second largest group (26%). About 44% of participants had a college degree or higher, and 37% reported annual household incomes between $50 000 and $99 999. At baseline visit 3 of this study, 49% were in early perimenopause, and 18% were postmenopausal; also, one-third were overweight, with 28% categorized as obese. Most were non-smokers, over half reported low alcohol consumption, and 18% had depression. Participants resided in neighborhoods with a mean population density of 3,924.3 people per square mile (SD = 4,933.2) (table 1).

**Table 1.** Characteristics of the study population (*N* = 2008) at visit 3, SWAN cohort, 1999–2017.

Characteristics		*n* (%)	
Age in years (mean (SD))		49.9 (3.1)	

Race and ethnicity	African American	516 (25.7)	
	Chinese	209 (10.4)	
	Hispanic	140 (7.0)	
	Japanese	231 (11.5)	
	White	912 (45.4)	

Education	High school	457 (22.8)	
	Some college	657 (32.7)	
	College or higher	875 (43.6)	
	Unknown	19 (0.9)	

Income (USD per annum)	Less than $19 999	191 (9.5)	
	$20 000–49 999	487 (24.3)	
	$50 000–99 999	732 (36.5)	
	$100 000 or more	437 (21.8)	
	Unknown	161 (8.0)	

Body mass index (BMI) kg m^−2^	BMI < 25 (normal)	730 (36.4)	
	BMI ⩾ 25 & BMI < 30 (overweight)	659 (32.8)	
	BMI ⩾ 30 (obese)	557 (27.7)	
	Unknown	62 (3.1)	

Menopausal status	Premenopausal	201 (10.0)	
	Early menopausal	982 (48.9)	
	Late menopausal	176 (8.8)	
	Postmenopausal	365 (18.2)	
	Unknown	284 (14.1)	

Current smoking status	Non-smoker	1705 (84.9)	
	Smoker	257 (12.8)	
	Unknown	46 (2.3)	

Alcohol consumption	Low	1060 (52.8)	
	Medium	492 (24.5)	
	High	405 (20.2)	
	Unknown	51 (2.5)	

Depression	No	1624 (80.9)	
	Yes	364 (18.1)	
	Unknown	20 (1.0)	

Population density people/mile^2^ (mean (SD))		3924.3 (4933.2)	

Abbreviations: SD-Standard Deviation; USD-United States Dollar.

*Note:*Percentage may not sum to 100% due to rounding.

The distributions of biomarkers across 6 visits and 5 categories of menopausal status reflect physiological and temporal variations within the SWAN cohort (table 2). Across visits, the median and IQR values of biomarkers varied, with patterns influenced both by time and menopausal status, for example, TC tended to increase during later visits, whereas hs-CRP showed relatively stable distributions over time. Biomarker levels, including inflammatory markers (e.g. hs-CRP and fibrinogen), were higher in post-menopause compared to the levels during pre-menopause and early perimenopause, which may partly reflect physiological changes associated with aging. Conversely, women had lower levels of lipids during pre-menopause, including TC and LDL. These biomarker values were consistent with other cohort studies across menopausal stages [40, 41].

**Table 2.** Distribution of cardio-inflammatory biomarkers by visits and menopausal status.

Total *N* = 8980	hs-CRP mg l^−1^ (*n* = 6196)	Endothelin-1 pg ml^−1^ (*n* = 3047)	Interleukin-6 pg ml^−1^ (*n* = 2682)	Cholesterol mg dl^−1^ (*n* = 7930)	HDL mg dl^−1^ (*n* = 7905)	LDL mg dl^−1^ (*n* = 7696)	TG mg dl^−1^ (*n* = 7730)	tPA-ag ng ml^−1^ (*n* = 4556)	Fibrinogen mg dl^−1^ (*n* = 5183)
Median (IQR)	1.6 (3.9)	1.3 (0.5)	1.8 (1.8)	200.5 (47.9)	59.0 (21.1)	115.0 (40.8)	102.0 (67.1)	6.7 (4.2)	292.0 (124.0)

Visit									

03 (*n* = 1759)	1.7 (4.0)	1.1 (0.5)	1.7 (1.7)	195.0 (46.0)	58.0 (21.0)	111.0 (39.0)	101.0 (72.0)	7.1 (4.4)	267.0 (70.0)
05 (*n* = 1717)	1.7 (4.2)	1.2 (0.5)	1.8 (1.5)	198.0 (48.0)	58.0 (20.2)	114.0 (42.0)	104.0 (68.0)	6.9 (3.7)	267.0 (74.0)
07 (*n* = 1630)	1.5 (3.6)	1.2 (0.5)	1.7 (1.7)	204.0 (47.0)	58.0 (22.0)	119.0 (41.5)	104.0 (72.0)	6.1 (4.4)	267.0 (74.0)
09 (*n* = 899)	—	1.2 (0.5)	1.7 (1.7)	—	—	—	—	—	—
12 (*n* = 1653)	—	1.4 (0.6)	2.1 (1.9)	204.0 (50.0)	59.0 (22.0)	117.0 (41.0)	103.0 (65.5)	—	—
15 (*n* = 1322)	1.6 (3.7)	—	—	203.3 (47.0)	63.7 (21.6)	114.6 (35.3)	101.7 (57.0)	—	447.0 (110.0)

Menopausal status									

Pre-menopausal (*n* = 307)	1.6 (3.8)	1.2 (0.4)	1.7 (1.9)	190.5 (36.0)	54.0 (20.0)	110.0 (38.0)	94.0 (70.5)	6.8 (4.1)	262.0 (66.0)
Early perimenopausal (*n* = 2086)	1.4 (3.5)	1.2 (0.5)	1.6 (1.5)	191.0 (45.0)	57.0 (19.0)	111.0 (38.0)	96.0 (63.0)	6.9 (4.0)	267.0 (66.0)
Late perimenopausal (*n* = 576)	1.5 (4.0)	1.2 (0.6)	1.9 (2.0)	207.5 (48.8)	59.0 (20.0)	121.0 (43.5)	105.5 (75.8)	7.1 (4.8)	273.0 (78.0)
Post menopausal (*n* = 5294)	1.7 (4.1)	1.3 (0.6)	1.9 (1.8)	204.3 (49.0)	60.0 (22.0)	117.0 (40.8)	104.3 (65.8)	6.5 (4.4)	356.0 (178.0)
Unknown (*n* = 717)	2.1 (4.1)	1.4 (0.4)	2.6 (2.1)	200.0 (45.8)	59.0 (24.0)	112.0 (42.2)	108.0 (75.0)	6.4 (3.9)	267.0 (70.0)

Abbreviations: hs-CRP-High-Sensitivity C-Reactive Protein, Cholesterol-Total Cholesterol; HDL-High Density Lipoprotein; LDL-Low Density Lipoprotein; TG-Triglycerides; tPA-ag-Tissue-Type Plasminogen Activator Antigen; IQR-Interquartile Range.

*Note:* N represents the total number of biomarker observations across all study visits, while n represents the number of observations with available data within each stratum.

**Figure 1.**
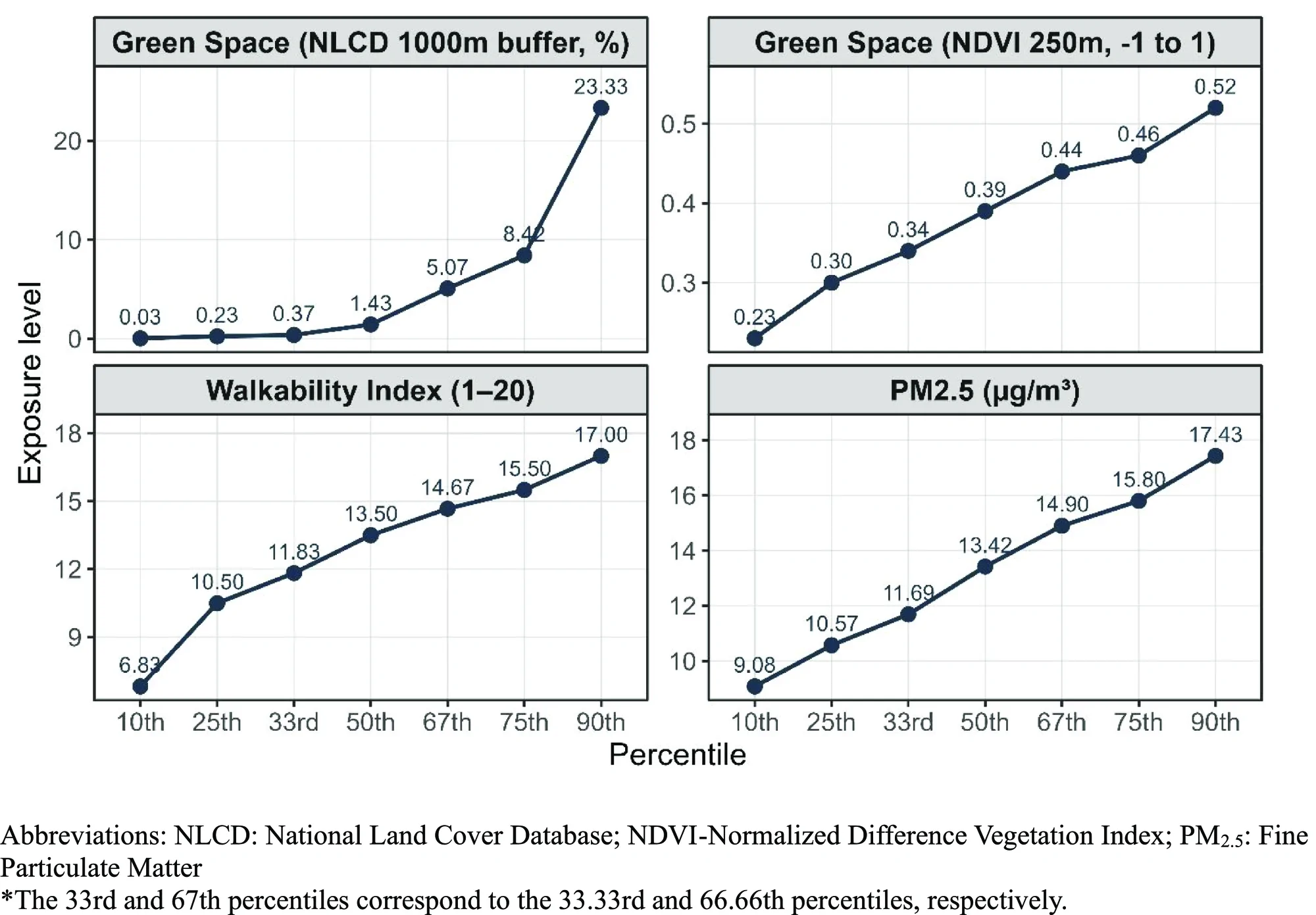
Percentile distribution of residential characteristics.

Figure 1 presents the distribution of residential land use and environmental characteristics over the study period. Green space by NLCD, weighted by residential history, shows a left-skewed distribution, with most participants residing in areas of relatively low greenness. The 33rd and 67th percentiles are 0.37% and 5.07% for green space by NLCD within a 1000 m buffer, and 0.34 and 0.44 for NDVI in the prior year. The corresponding percentiles for the walkability index are 11.83 and 14.67, and for PM_2_._5_, 11.69 and 14.90 *μ*g m ^−3^, capturing variability in these exposures.

We examined Spearman correlations among green space measures (NLCD 1000 m, 500 m, and NDVI 250 m), walkability, and PM_2_._5_ (supplementary figure S4). NLCD green space at 500 m and 1000 m were strongly correlated (*ρ* = 0.82), indicating spatial consistency across buffer sizes. NDVI showed moderate positive correlations with NLCD measures (*ρ* = 0.50–0.52), reflecting differences in greenness quantification across metrics. Walkability was inversely correlated with green space (*ρ* ≈ −0.31 to −0.49), with the strongest inverse association observed with NDVI, consistent with contrasting urban density and vegetation patterns. PM_2_._5_ demonstrated weak correlations with green space (*ρ* ≈ −0.12 to −0.05) and a weak positive correlation with walkability (*ρ* = 0.12).

**Figure 2.**
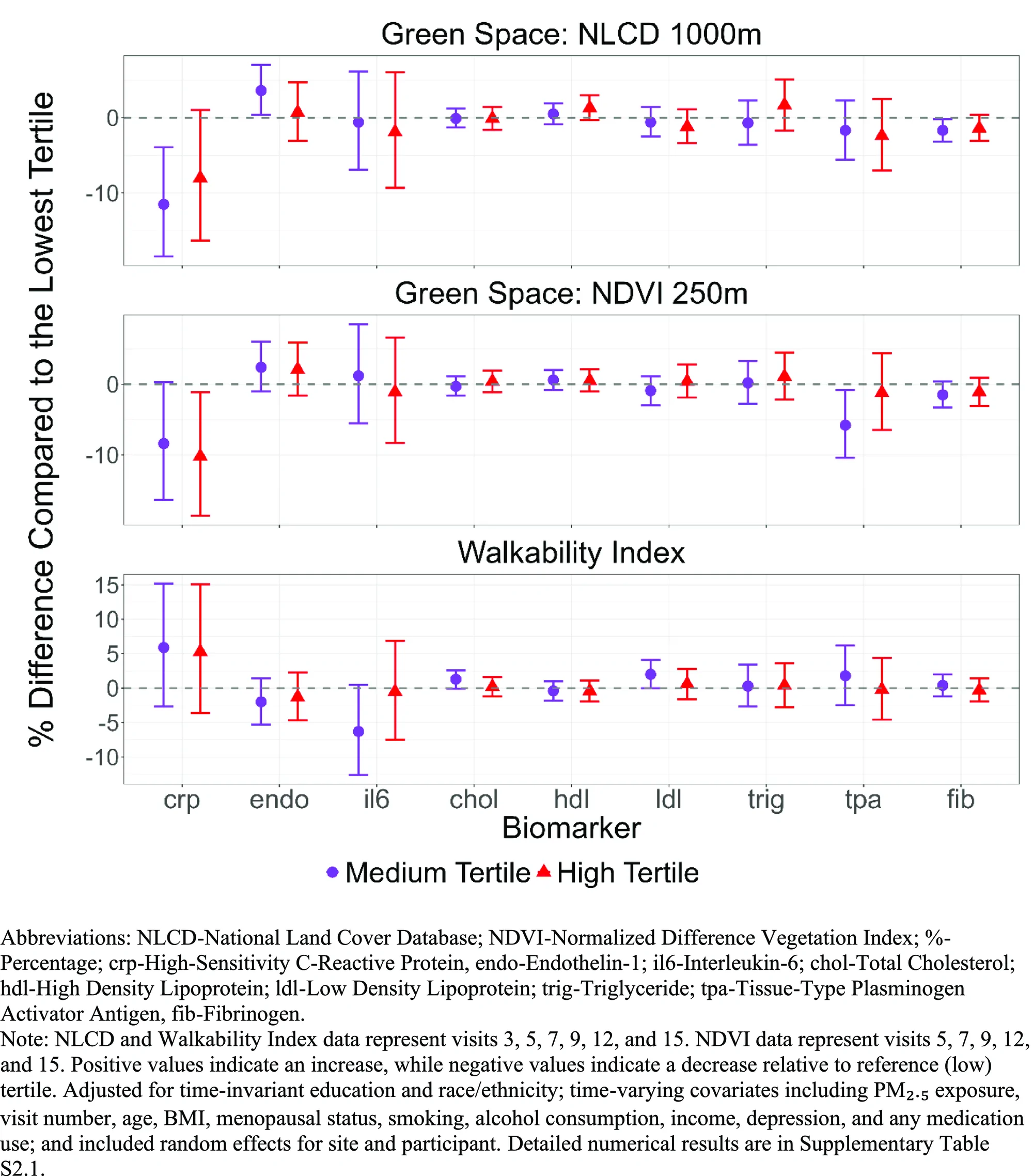
Association between residential green space (NLCD and NDVI), walkability, and cardio-inflammatory biomarkers.

The analysis of residential land use indicators and cardio-inflammatory biomarkers in the SWAN cohort revealed several key findings (figure 2). Compared to the low tertile (reference category), the medium exposure tertile of NLCD within a 1000 m buffer was associated with 11.5% lower (95% CI: −18.4, −3.9%) hs-CRP levels, while the high tertile was linked to an 8.0% reduction (95% CI: −16.3, 1.0%). Similarly, fibrinogen levels were 1.7% lower (95% CI: −3.2, −0.2%) in the medium tertile of greenness. In contrast, the medium greenness category was associated with a 3.6% higher (95% CI: 0.4, 7.0%) endothelin-1 levels. Compared to the lowest tertile, women in the highest green space tertile had 1.4% lower fibrinogen (95% CI: −3.1%, 0.4%), 0.7% higher endothelin-1 (95% CI: −3.1%, 4.7%), and 1.3% higher HDL (95% CI: −0.3%, 3.0%) levels.

Green space measured using NDVI showed that the medium tertile of NDVI-weighted greenness was associated with an 8.4% lower level of hs-CRP (95% CI: −16.4%, 0.3%), while the high tertile corresponded to a 10.2% lower level (95% CI: −18.6%, −1.1%). Medium NDVI greenness was also linked to 5.8% lower tPA-ag levels (95% CI: −10.4%, −0.8%).

For walkability, the medium tertile was associated with 6.3% lower IL-6 levels (95% CI: −12.6%, 0.5%), although the CI included the null. The same level of walkability was also linked to 2.0% higher LDL (95% CI: 0.0%, 4.1%) and 1.3% higher TC (95% CI: −0.1%, 2.6%). No significant associations were observed for other biomarkers.

### 3.1. Interaction analyses

**Figure 3.**
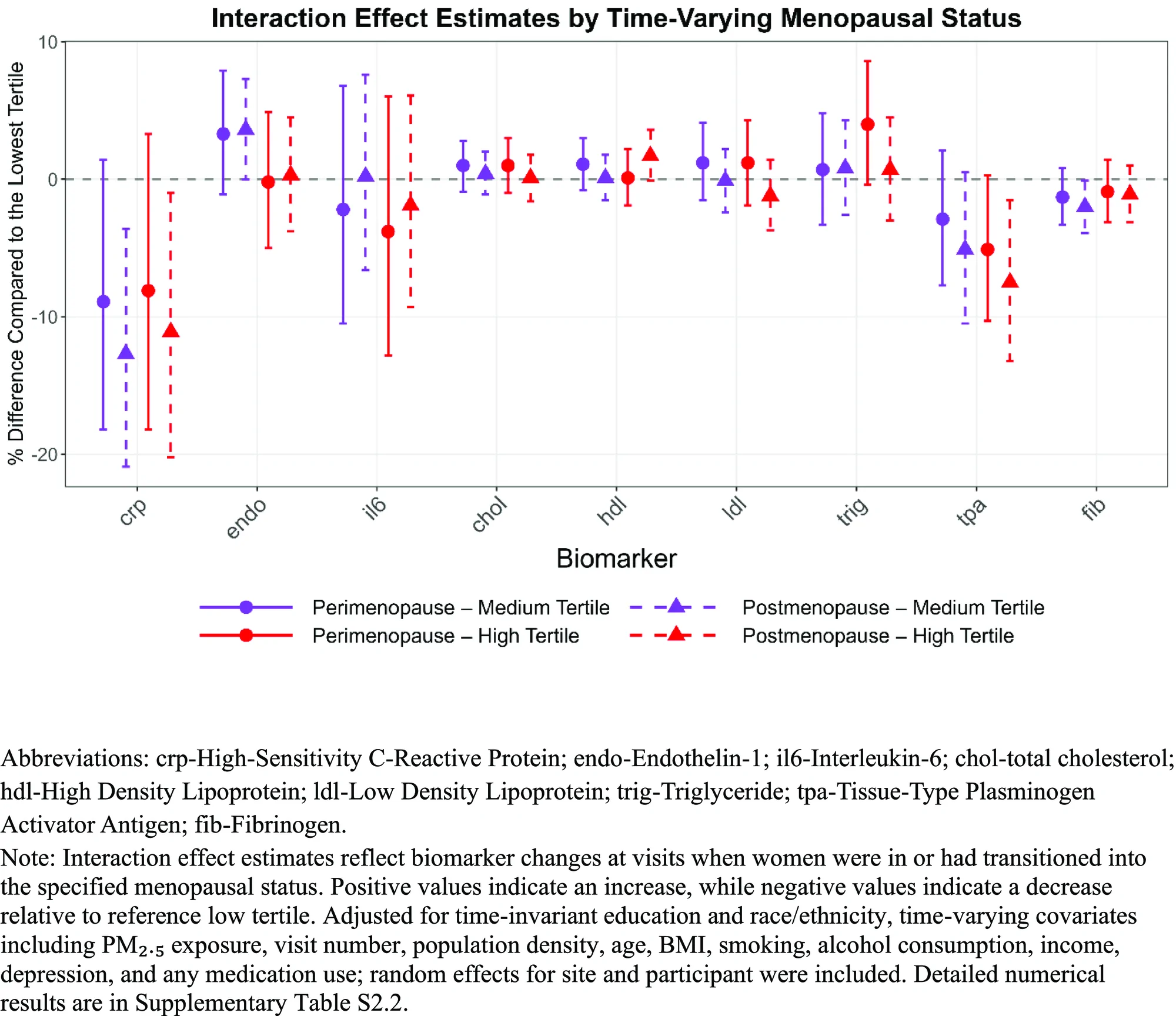
Interaction effect estimates of residential green space (NLCD 1000 m) on biomarkers across time-varying menopausal stages.

Results from the interaction analysis (figure 3) showed that associations between residential green space and cardiometabolic biomarkers varied by menopausal status. Specifically, hs-CRP indicated a greater reduction with green space exposure in women who had transitioned to or were in postmenopausal status, whereas perimenopausal women exhibited greater variability. In women who transitioned into postmenopausal period, high green space exposure was associated with higher HDL levels and a weak, non-significant attenuated association with triglycerides. In the same group, endothelin-1 was higher in medium green space tertile. No consistent patterns were observed for other biomarkers. It should be noted, however, that the global test for interaction did not indicate significant effect modification by menopausal status except HDL (*p*-value = 0.03, supplementary table S1).

Across all BMI strata, CRP showed consistent inverse associations with medium and high green space exposure with the strongest reductions in the obese group for the high exposure tertile, based on visit-specific BMI for each participant (supplementary figure S5). A suggestive interaction was observed for tPA-ag with high green space exposure in the normal BMI group, though not statistically significant (*p*-value = 0.07; table S1).

### 3.2. Sensitivity analyses

In sensitivity analyses adjusting for walkability, associations between green space (NLCD and NDVI) and biomarkers remained consistent with the primary analysis. Levels of hs-CRP showed reduction with both greenness metrics in the medium and high tertile categories. Medium NLCD greenness was also associated with lower fibrinogen levels, while medium NDVI was linked to reduced tPA-ag levels. In contrast, endothelin-1 was high for medium NLCD, while lipid biomarkers showed no notable changes (supplementary figure S6).

To emphasize the cohort’s longitudinal nature and minimize bias from incomplete follow-up, a sensitivity analysis was conducted among participants with data across all visits (supplementary figure S7). Medium greenness (NLCD 1000 m) was linked to lower tPA-ag levels. Despite reduced sample sizes, the trends in biomarker changes were similar to those in the primary analyses, suggesting the robustness of exposure-outcome relationships. The sensitivity analysis using the 500 m buffer NLCD green space exposure yielded similar findings, except for gain in HDL levels. Sequential adjustment analyses indicated modest attenuation of associations after exclusion of BMI, particularly for hs-CRP and tPA-ag, whereas estimates for fibrinogen were minimally affected. These findings suggest partial overlap between adiposity-related pathways and inflammatory biomarkers, while the primary associations remained directionally consistent (supplementary figure S8).

In another sensitivity analysis using continuous exposure specifications (per IQR increase), associations were directionally consistent with the primary tertile-based models. Higher greenness (NLCD 1000 m and NDVI 250 m) was associated with lower levels of inflammatory biomarkers, including hs-CRP, fibrinogen, and tPA-ag, while lipid biomarkers showed minimal changes. Walkability showed patterns similar to those observed in the categorical analyses (supplementary figure S9).

All numerical results presented in figures in the manuscript are provided in supplementary table S2 (S2.1–S2.5).

## 4. Discussion

This study is among the few longitudinal investigations examining the relationship between residential land use, including green space and walkability, and cardio-inflammatory biomarkers in midlife women over nearly two decades, a life stage with increased cardiovascular vulnerability. Higher residential green space exposure (NLCD 1000 m and NDVI 250 m) was associated with lower levels of inflammatory biomarkers hs-CRP, fibrinogen, and tPA-ag, while associations with lipid biomarkers were minimal. Notably, despite NLCD being measured every five years and NDVI reflecting one year, their consistent associations highlight the robustness of our findings. The use of different buffer sizes (NLCD 1000 m and NDVI 250 m) reflects the distinct spatial properties of these metrics and allowed us to capture both broader neighborhood land cover context (approximately a 10–15 minute walk) and proximal residential greenness (approximately a 3–5 minute walk) [42].

We examined walkability as both an independent predictor and a covariate alongside green space, allowing us to assess how walkability and green space independently and collectively influence biomarker levels. Adjusting for PM_2_._5_, higher walkability tertile was associated with higher LDL and TC, consistent with the urban health paradox, where walkability promotes activity but increases exposure to environmental and psychosocial stressors [10]. To disentangle the effects of walkability from urban environmental stressors, we conducted sensitivity analyses adjusting for it as a distinct built environment factor in the model. While some lipid biomarker associations were attenuated, the primary associations of green space exposure with hs-CRP, fibrinogen, and tPA-ag remained largely unchanged, suggesting the observed patterns of association with inflammation may not be solely mediated by walkability.

Several studies across diverse populations suggest that green space and favorable land use, including high walkability, offer cardioprotective effects by improving air quality exposure, reducing stress, and promoting physical activity [8, 43, 44]. However, the evidence remains inconsistent. A systematic review linked greater greenspace access (NDVI-measured or self-reported) to improved cardiovascular and metabolic health, but noted substantial heterogeneity and many null findings [45]. The Dutch birth cohort (adolescents aged 12 and 16 years) utilizing NDVI 300 m and 3000 m found no significant associations with cardiometabolic health, suggesting age may be a key factor in the differing findings [46]. While some studies have not found associations between green space and reduced cardiovascular mortality [47, 48], our analysis suggests subclinical benefits through lower inflammatory biomarkers in midlife women. Variations in population, outcomes, and gender-specific factors may explain these differences. Inconsistencies may also stem from social inequities, exposure misclassification, and differences in spatial resolution and land use classification [49].

Environmental exposures such as green space, walkability, and air pollution can link with cardiovascular health through multiple biological mechanisms. Vegetation can reduce pollutant concentrations by enhancing deposition of PM_2_._5_ and volatile organic compounds, thereby potentially lowering systemic inflammation and endothelial dysfunction [24]; green space has also been associated with lower IL-6, TNF-*α*, and C-reactive protein (CRP), possibly through pathways involving pollution reduction, stress mitigation, and physical activity. In our data, green space measures were mostly inversely correlated with PM_2_._5_, suggesting that greener neighborhoods may, on average, experience slightly lower particulate pollution. However, these correlations were modest, and the relationship between vegetation and air pollution is complex. The effects of greenery depend on species, canopy density, and spatial configuration; in some urban settings vegetation may enhance pollutant removal, while dense tree canopies can limit airflow and trap pollutants [12, 50]. Such contextual variation may contribute to the relatively small correlations observed in our cohort.

Air pollution, particularly PM_2.5_, induces oxidative stress and endothelial dysfunction, stimulating the release of IL-6, CRP, and endothelin-1, and other vasoconstrictors [18, 46, 51]. IL-6-driven increases in fibrinogen, plasminogen activator inhibitor-1 (PAI-1), and CRP further accelerate CVD [52–54]. Beyond inflammation, neuroimmune modulation, lipid imbalances, including elevated LDL and TG and reduced HDL, contribute to vascular remodeling, plaque instability, and thrombosis [55, 56]. Chronic urban stress activates the sympathetic nervous system and disrupts the hypothalamic-pituitary-adrenal axis, further exacerbating endothelial injury [55, 57]. These mechanisms may help explain the observed associations between residential green space and lower inflammatory biomarker levels, while the potential role of lipid metabolism in this relationship warrants further investigation.

Inflammation is a key driver of CVD in adults, especially during the menopausal transition in midlife women [58]. In our study, long-term reductions in inflammatory markers, including hs-CRP, IL-6, and fibrinogen, with greater green space exposure were consistent with prior research [43, 59]. However, reductions in dyslipidemia markers such as TC, LDL, and TG, as well as increases in HDL, were not observed as expected. Given HDL’s role in reverse cholesterol transport and its anti-inflammatory effects, higher HDL levels are typically linked to reduced systemic inflammation and improved lipid regulation [60]. The urban nature of our study sites may have influenced this outcome, as high pollutant levels in densely populated areas could offset lipid benefits expected from walkability and physical activity. Additionally, elevated levels of endothelin-1, a vasoconstrictor involved in endothelial dysfunction during inflammatory conditions were observed [61–63]. Previous studies have linked acute air pollution exposures, particularly diesel exhaust, to higher endothelin-1 levels, suggesting that residual confounding by atmospheric pollutants may have influenced this finding in our study [51, 64].

Beyond environmental exposures, our findings underscore the importance of studying women undergoing the menopausal transition, as menopausal status emerged as a more relevant potential effect modifier than BMI. Both factors plausibly influence systemic inflammation and cardiometabolic regulation, though estrogen decline during menopausal transition may have stronger effects. This hormonal shift increases central adiposity, systemic inflammation, and adverse lipid changes, heightening cardiovascular risk [17, 37]. Higher BMI further exacerbates these effects, as visceral fat promotes inflammation via pro-inflammatory cytokines (IL-6, TNF-*α*), contributing to insulin resistance, dyslipidemia, and hypertension [38]. Given the role of inflammation and lipid dysregulation in disease progression, green space may help mitigate these risks. While our findings do not provide strong statistical evidence of effect modification for most biomarkers, trends in interaction analyses suggest potential biological interactions. These associations were most evident in the women who were or transitioned into post-menopausal period, with hs-CRP showing reductions with both high and medium green space levels, and higher HDL and lower triglycerides observed only with greater green space. These findings warrant further investigation in larger cohorts with additional covariate adjustments, such as endogenous hormone levels, to better understand potential interactions.

Sensitivity analyses restricted to participants with biomarker data available across all visits helped minimize potential biases due to incomplete follow-up while reinforcing the longitudinal nature of the study. The results yielded similar associations, though reduced sample size may have contributed to the wider CIs for some biomarker changes.

### 4.1. Strengths and limitations

This study has several strengths, including its longitudinal design with time-weighted exposure metrics, which provides a more comprehensive assessment of green space exposure compared to cross-sectional studies. Few longitudinal studies have examined residential land use and cardio-inflammatory biomarkers in midlife women, as most focus on other populations. Prior research on perceived green space quality emphasizes early-life health benefits [65], while our study extends this inquiry to midlife—a period of heightened cardiometabolic risk. By incorporating long-term residential histories, we enhance exposure assessment, reducing misclassification and better capturing the chronic effects of green space. Additionally, assessing both NDVI and NLCD as green space predictors enhances the analysis by offering complementary insights into greenness: NLCD enhances the analysis by categorizing land cover types, capturing long-term land use patterns, while NDVI provides a dynamic, fine-grained measure of vegetation density with frequent updates that reflect seasonal and short-term changes.

Another strength of this study is the sensitivity analysis using a 500 m circular buffer, which yielded findings consistent with the primary analysis using a 1000 m NLCD buffer. The 500 m and 1000 m buffers capture both walkable and broader neighborhood greenspace, enabling assessment of its impact on cardiometabolic health via physical activity, well-being, and environmental factors [42, 66]. Furthermore, SES influences the association between green space and cardiovascular health, as lower-income neighborhoods often lack quality green spaces and face greater exposure to environmental toxins, increasing CVD risk [22, 67]. Our study adjusted for SES indicators, such as income and education, to mitigate confounding from neighborhood disparities. Additionally, SWAN’s multiethnic, multicenter design enhances internal validity by ensuring adequate representation of multiple racial/ethnic groups across diverse urban communities, allowing meaningful within-group comparisons in midlife women.

Despite these strengths, some limitations remain. While our models adjusted for age, disentangling the effects of chronological aging from ovarian aging associated with the menopausal transition remains challenging. Age is a key confounder in this context, and its influence cannot be fully isolated. Limitations in green space measurement may contribute to exposure misclassification, as NLCD data may miss short-term changes and NDVI can be affected by cloud cover. Moreover, these metrics quantify the amount and density of vegetation but do not reflect spatial configuration, accessibility, or functional characteristics of green space (e.g. centralized parks versus dispersed vegetation). Our measures reflect residential proximity to surrounding vegetation and do not account for accessibility, utilization, or time spent in specific green spaces, such as parks, at an individual level. Although we adjusted for PM_2.5_, residual confounding may persist as the health effects of other pollutants, such as ozone and nitrogen dioxide, were not fully accounted for. Biomarker data availability varied across study visits, limiting sample size for some analyses. For example, tPA-ag levels were only available through visit 7, while NDVI data were only available from visit 5 onward, restricting the ability to fully assess long-term associations. We also acknowledge that individual-level factors, such as diet and nutrition, may influence cardiometabolic biomarkers and could contribute to variability in the observed associations, despite adjustment for related socioeconomic and behavioral factors. Lastly, although SWAN includes diverse racial/ethnic groups across multiple U.S. metropolitan areas, findings should be interpreted in the context of similar urban midlife populations, as environmental and infrastructure characteristics may differ across settings. In addition, we did not account for time–activity patterns, such as work or travel, which may influence actual green space exposures beyond residential location.

## 5. Conclusion

This study finds that residential green space exposure is associated with lower levels of some inflammatory and hemostatic biomarkers in midlife women, with potential implications for cardiovascular health while benefits of walkability remain debatable in the urban settings. There is some indication of effect modification by menopausal status warranting further investigation. Future studies using quasi-experimental designs to evaluate newly implemented urban greening interventions could provide stronger evidence for causal pathways and help disentangle the effects of green space from other environmental and social determinants of health. Integrating green infrastructure into urban planning while minimizing unintended consequences, such as gentrification and increased pollutant exposure, will be essential for fostering healthier urban environments and reducing the burden of chronic disease.

## Data Availability

SWAN provides access to public use datasets that include data from SWAN screening, the baseline visit and follow-up visits (https://agingresearchbiobank.nia.nih.gov/). To preserve participant confidentiality, some, but not all, of the data used for this manuscript are contained in the public use datasets. A link to the public use datasets is also located on the SWAN web site: http://www.swanstudy.org/swan-research/data-access/. Investigators who require assistance accessing the public use dataset may contact the SWAN Coordinating Center at the following email address: swanaccess@edc.pitt.edu. Supplementary Material available at https://doi.org/10.1088/2752-5309/ae61e7/data1.
